# A systematic review on multiparametric MR imaging in prostate cancer detection

**DOI:** 10.1186/s13027-017-0168-z

**Published:** 2017-10-30

**Authors:** Roberta Fusco, Mario Sansone, Vincenza Granata, Sergio Venanzio Setola, Antonella Petrillo

**Affiliations:** 1 Radiology Unit, “Dipartimento di supporto ai percorsi oncologici Area Diagnostica, Istituto Nazionale Tumori - IRCCS - Fondazione G. Pascale”, Via Mariano Semmola, Naples, Italy; 2 Department of Electrical Engineering and Information Technologies, University “Federico II” of Naples, Via Claudio, Naples, Italy

**Keywords:** Prostate cancer, Magnetic Resonance Spectroscopic Imaging, Diffusion Weighted Imaging, Dynamic Contrast Enhanced Imaging, Coil setting

## Abstract

**Background:**

Literature data suggest that multi-parametric Magnetic Resonance Imaging (MRI), including morphologic T2-weigthed images (T2-MRI) and functional approaches such as Dynamic Contrast Enhanced-MRI (DCE-MRI), Diffusion Weighted Imaging (DWI) and Magnetic Resonance Spectroscopic Imaging (MRSI), give an added value in the prostate cancer localization and local staging.

**Methods:**

We performed a systematic review of literature about the role and the potentiality of morphological and functional MRI in prostate cancer, also in a multimodal / multiparametric approach, and we reported the diagnostic accuracy results for different imaging modalities and for different MR coil settings: endorectal coil (ERC) and phased array coil (PAC). Forest plots and receiver operating characteristic curves were performed. Risk of bias and the applicability at study level were calculated.

**Results:**

Thirty three papers were identified for the systematic review. Sensitivity and specificity values were, respectively, for T2-MRI of 75% and of 60%, for DCE-MRI of 80% and of 72%, for MRSI of 89% and of 69%, for combined T2-MRI and DCE-MRI of 87% and of 46%, for combined T2-MRI and MRSI of 79% and of 57%, for combined T2-MRI, DWI and DCE-MRI of 81% and of 84%, and for combined MRSI and DCE-MRI of 83% and of 83%. For MRI studies performed with ERC we obtained a pooled sensitivity and specificity of 81% and of 66% while the pooled values for MRI studies performed with PAC were of 78% and of 64%, respectively (p>0.05 at McNemar test). No studies were excluded from the analysis based on the quality assessment.

**Conclusions:**

ERC use yielded no additional benefit in terms of prostate cancer detection accuracy compared to multi-channel PAC use (71% versus 68%) while the use of additional functional imaging techniques (DCE-MRI, DWI and MRSI) in a multiparametric MRI protocol improves the accuracy of prostate cancer detection allowing both the early cure and the guidance of biopsy.

## Background

Approximately 180,890 new prostate cancers are expected in 2016 in the USA [1]. The most well-recognized risk factors for the development of prostate cancer are old age, family history, testosterone, ethnic origin, environment and genetic factors [2]. One such potential environmental factor which has gained a great deal of recent attention is the development of chronic inflammation in the prostate due to a number of potential causes including infections, dietary factors, hormonal changes and/or other unknown environmental exposures [2]. The exact mechanisms of the progression of prostate gland into a cancer are not well characterized. The growing epidemiological studies have suggested that prostate tissue is prone to sexually transmitted infection with several viruses having oncogenic potential such as polyomaviruses, human papillomaviruses, and members of the herpes virus family [3–8].

Early detection of prostate cancer can lead to a complete cure [2–10]. Mainly, the diagnosis of prostate cancer is based on the results of ultrasonography (US)-guided transrectal biopsy. A random biopsy was typically performed to overcome the ultrasonography limits in prostate cancer detection and localization. However, a random biopsy has several disadvantages such as an increase in complications because of the unnecessary sampling of normal prostate tissue while cancer that is located outside the routine biopsy site may be missed. In addition, there may be difficulties in determining the site of a previous biopsy when repeating biopsy in a patient with a previous negative result and continuous high prostate-specific antigen (PSA) levels. A more advanced imaging modality is needed for accurate detection and localization of prostate cancer, as well as guidance of biopsy.

Magnetic resonance imaging (MRI) has been used to evaluate prostate anatomy and pathologies: it provides high-resolution images of the prostate and surrounding structures. T2-weighted magnetic resonance imaging (T2-MRI) with endorectal coil (ERC) and pelvic phased array coil (PAC) has been widely used for pre-treatment work-up of prostate cancer [2–17]. However, T2-MRI has substantial restrictions for depicting cancer in the transitional and central zones, because both cancer and normal tissues have low signal intensity. In addition, low signal intensity may be seen in the peripheral zone on T2-MRI in case of some noncancerous abnormal conditions, such as inflammation, biopsy related haemorrhage, post–radiation therapy fibrosis [15–22].

Other MRI modalities might be used to increase the diagnostic accuracy in prostate cancer detection and localization such as Magnetic Resonance Spectroscopic Imaging (MRSI), Dynamic Contrast Enhanced MR Imaging (DCE-MRI) and Diffusion Weighted Imaging (DWI).

**Fig. 1:**
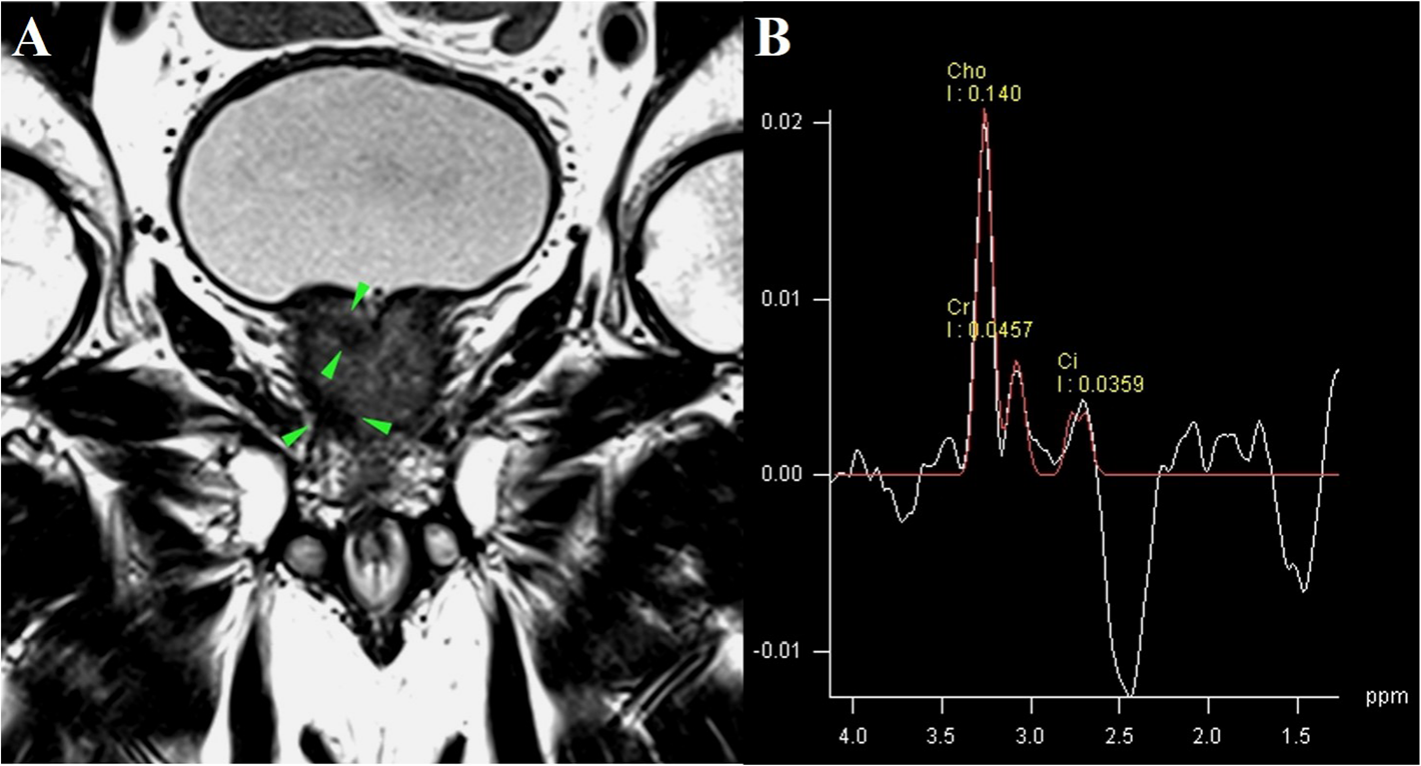
MRSI (**a**) in prostate cancer with correspondent metabolite spectrum (**b**)

MRSI measures metabolite levels in the tissue such as choline (Ch), citrate (Cit), creatine (Cr), and various polyamines (spermine, spermidine, and putrecine) (Fig. 1). Prostate cancer usually shows an increased concentration of Ch and reduction of Cit and polyamines. Several studies have shown the benefit of adding MRSI to MRI in the evaluation of prostate cancer [2–13]. Studies have shown the ability of MRSI to improve the cancer detection rate in patients with high PSA [2]; moreover, MRSI has shown itself promising in assessment of cancer aggressiveness [12, 13]. The accuracy of MRSI is generally accepted but it is important to avoid magnetic field distortions that may influence the MR spectrum. Moreover, MRSI requires a long acquisition time and more expertise; MRSI does not directly depict the peri-prostatic area and is often affected by artefacts.

**Fig. 2:**
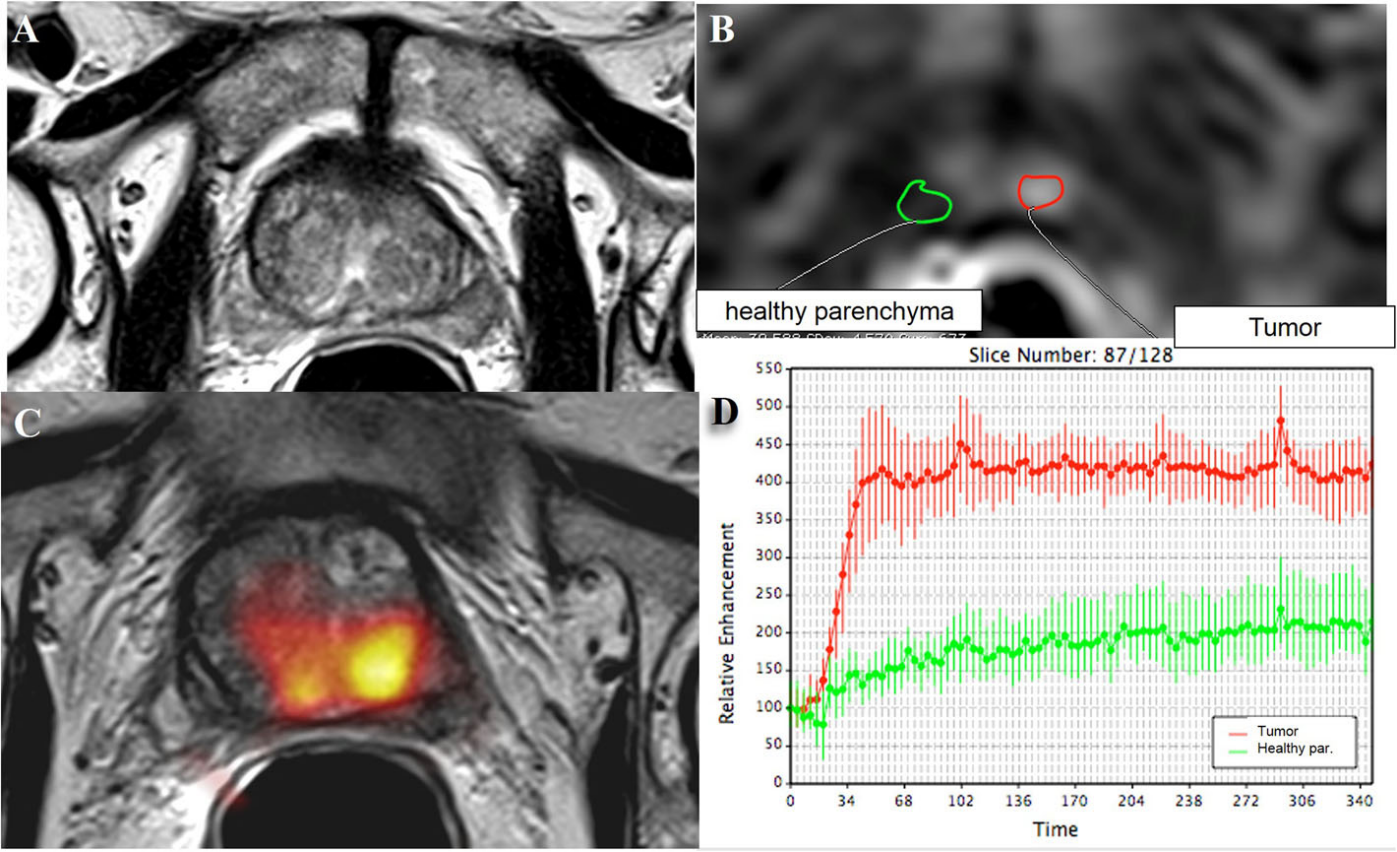
DCE-MRI (**a**, **b**, **c**) with time intensity curve (**d**) for tumor area and healthy parenchyma area

**Fig. 3:**
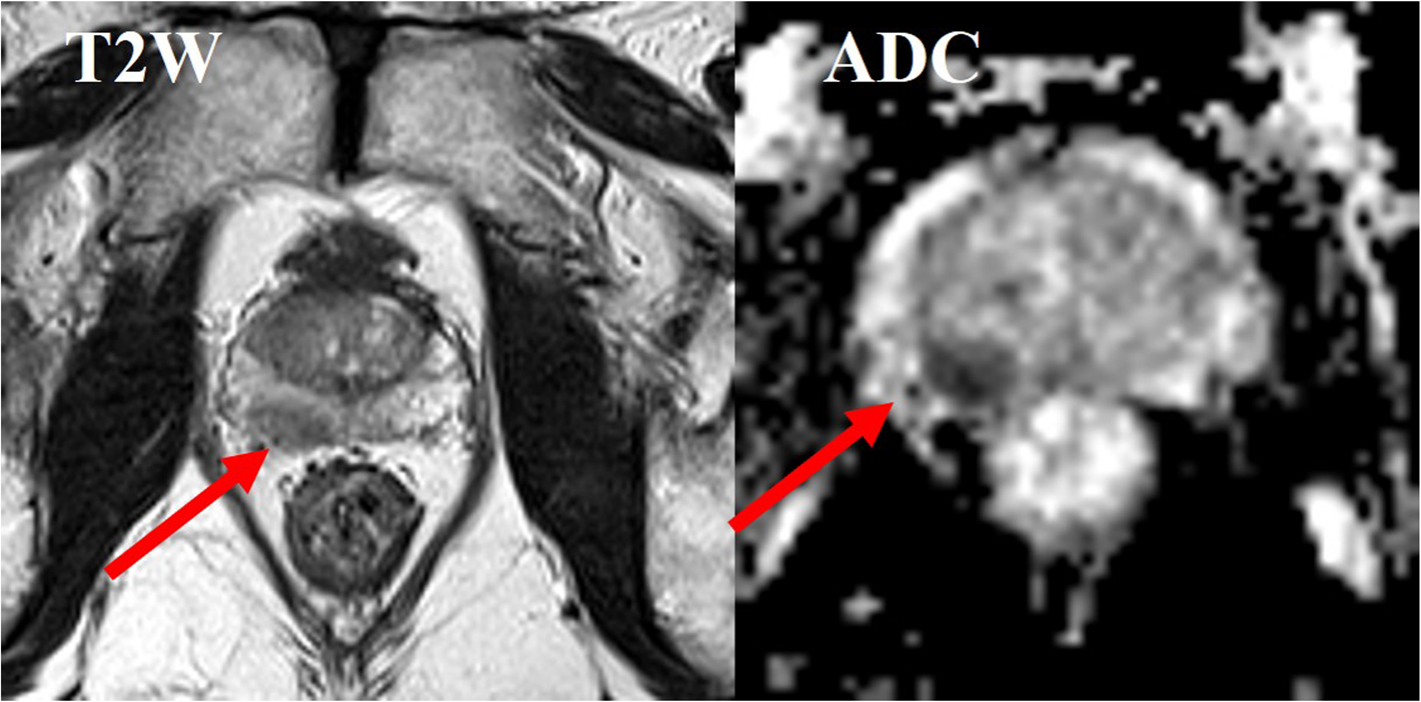
T2-MRI with apparent diffusion coefficient map of DWI

Dynamic contrast enhanced MRI (DCE-MRI) has been developed for the assessment of perfusion parameters allowing to differentiate cancer from normal tissue [19, 23–29] (Fig. 2). The advantages of this technique include the direct or indirect depiction of tumour vascularity; however, because of overlap of enhancement pattern with benign conditions such as prostatitis in the peripheral zone and benign prostate hyperplasia nodules in the transition zone, DCE MRI is not considered as a dominant imaging sequence in prostate cancer detection. DCE-MRI is often applied as an adjunct to T2-MRI and Diffusion weighted imaging (DWI) findings in multiparametric MRI approach [14]. DWI assesses the restriction of diffusion and the reduction of apparent diffusion coefficient (ADC) values in cancerous tissue [30–33] (Fig. 3). Despite significant differences in the mean ADC values between cancerous and normal tissues, individual variability may decrease ADC diagnostic accuracy for prostate cancer detection and localization [30–41].

Early promising data suggest that multimodal, also referred to as multiparametric MRI, combining several MRI modalities such as morphologic T2-MRI sequences and DCE-MRI or DWI and/or MRSI (functional MRI modalities), may be of additional value for the localization of prostate cancer and guidance of biopsy [12, 13, 41–61]. The combined use of T2-MRI and functional MRI modalities (DCE-MRI, DWI and/or MRSI) has been shown to improve cancer localization [53, 54] and cancer volume measurement [47] in the peripheral zone. Petrillo et al. [12] demonstrated that a combined score of morphological T2-MRI, DWI and MRSI had: (i) the highest sensitivity (0.84) and negative predictive value (0.93) in prostate cancer detection; (ii) a significant correlation with Gleason score; and (iii) a statistically different median value between significant and not significant Gleason score.

A comprehensive evaluation in which both morphological and functional MRI modalities (DCE-MRI, DWI, MRSI) are used with an understanding of their particular advantages and disadvantages could be of help. In this manuscript, the authors will provide a systematic review of literature contributes about the role and the potentiality of T2-MRI, DCE-MRI, DWI, MRSI and their combinations in a multiparametric approach for prostate cancer detection.

## Methods

The review is the result of autonomous studies without protocol and registration number.

### Search Criterion

Several electronic databases were searched: PubMed (US National Library of Medicine, http://www.ncbi.nlm.nih.gov/pubmed), Scopus (Elsevier, http://www.scopus.com/), Web of Science (Thomson Reuters, http://apps.webofknowledge.com/) and Google Scholar (https://scholar.google.it/). The following search criteria have been used: “prostate cancer” AND “T2-weighted magnetic resonance imaging”, “prostate cancer” AND “conventional magnetic resonance imaging”, “prostate cancer” AND “dynamic contrast enhanced magnetic resonance imaging”, “prostate cancer” AND “diffusion weighted magnetic resonance imaging”; “prostate cancer” AND “magnetic resonance spectroscopy imaging”, “prostate cancer” AND “multimodal imaging”, “prostate cancer” AND “multi-parametric imaging”, “prostate cancer”. The search covered the years from 2000 through 2016. Moreover, the reference lists of the found papers were analysed for papers not indexed in the electronic databases.

All titles and abstracts were analysed by two independent reviewers and exclusively the studies reporting MRI, DCE-MRI, DWI, MRSI and/or their combinations results in the prostate cancer detection and localization have been included.

If not otherwise stated, all the studies reviewed herein fulfill the following criteria: 1) English language; 2) thorough clinical characterization of the patients with prostate cancer studied by means MRI, DCE-MRI, DWI, MRSI and/or their combination and exclusion of studies using other diagnostic techniques; 2) articles, reviews and studies that did not present data about specificity, sensitivity, positive and negative predictive value of tests treated were excluded; 3) reviews, general overview articles and congress abstracts were excluded. There was no a minimum number of patients as an inclusion criteria due to the small number of studies for each imaging modality. Information extracted from each study included title, authors, year of publication, sample size, diagnostic modality, true and false positives number, true and false negatives number.

In this review, multimodal or multi-parametric MRI was considered as the combination of two diagnostic modalities. The combinations of two test can means: only one of the tests has to be positive for the result of the combination to be considered positive (indicated with “OR”), or all tests in the combination have to be positive before the result for the combination is considered positive (indicated with “AND”).

### Data Analysis

Review Manager (version 5.2) was used to perform data analysis for systematic review.

True and false positives number, true and false negatives number for each paper were collected and used to obtain the forest plots reporting the sensitivity, specificity values and relative 95% confidence intervals (CIs). Receiver operating characteristic (ROC) curves were also performed.

We assessed the risk of bias and the applicability at study level using the validated Quality Assessment of Diagnostic Accuracy Studies (QUADAS-2) scoring system. Four domains are scored: (1) patient selection; (2) index test, which describes the test being studied and how it was conducted and interpreted; (3) reference standard; and (4) flow and timing, which describe the flow of patient inclusion and exclusion and the interval between the index test and the reference standard. The quality assessment was performed by two independent reviewers. Any disagreements were resolved by discussion with a third reviewer.

## Results

**Fig. 4:**
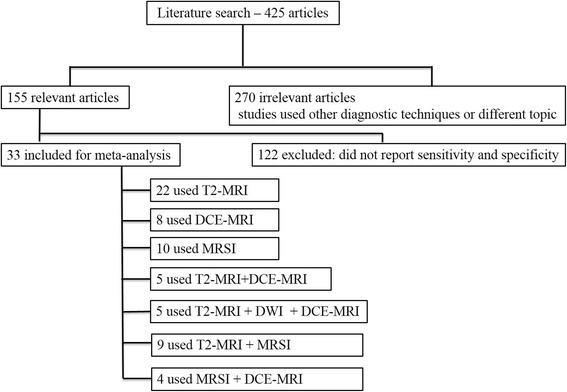
Included and excluded studies in systematic review

By using the search terms described earlier, we identified 425 studies from 2000 through 2016. 172 studies used other diagnostic techniques than MRI, DCE-MRI, DWI, MRSI and multimodal / multi-parametric imaging as a combination of two or more MRI modalities, 98 have different topic; 122 did not have sufficient data (did not report sensitivity and specificity). 33 studies remained for inclusion in our systematic review (Fig. 4).

Table 1 shows studies and participants number for each diagnostic modality. Table 2 reports studies characteristics. No study reporting the accuracy of MRSI combined with DWI was found.

**Table 1:** Number of studies and participants for each diagnostic modality

Diagnostic modality	Studies	Participants
T2-MRI	22	2294
MRSI	10	618
DCE-MRI	8	1445
T2-MRI combined with MRSI	12	407
T2-MRI combined with DCE-MRI	5	313
T2-MRI combined with DWI and DCE - MRI	5	2121
MRSI combined with DCE – MRI	4	394

**Table 2:** Studies characteristics. All studies used as a reference standard histopathological assessment of biopsied tissue obtained by TRUS

Authors	Modality	N. Patients	ERC versus PAC	MR Scanner TESLA Intensity
Amsellem-Ouazana et al. 2005	T2, MRSI, T2 or MRSI	42	ERC	1.5T
Babaian et al. 2000	T2	38	PAC	1.5T
Beyersdorff et al. 2002	T2	38	ERC	1.5T
Bhatia et al. 2007	MRSI, T2 and MRSI	21	ERC	1.5T
Bloch et al. 2007	T2, DCE, T2 and DCE	32	ERC	1.5T
Bloch et al. 2012	T2 and DCE	108	ERC	3T
Cheikh et al. 2009	T2, DCE, T2 or DCE	93	PAC	1.5T
Cirillo et al. 2008	T2, MRSI, T2 or MRSI	54	ERC	1.5T
Destefanis et al. 2009	T2 or MRSI	26	ERC	1.5T
Franiel et al 2011	T2, T2 or MRSI, T2 or DCE	54	ERC	1.5T
Haider 2007	T2, T2 and DCE	392	ERC	1.5T
Iwazawa et al. 2011	T2 or DWI or DCE	1424	PAC	1.5T
Kim et al. 2005	T2, DCE	954	PAC	1.5T
Kitajima et al. 2010	T2 or DWI or DCE	424	PAC	3T
Lattouf et al. 2007	T2, DCE, T2 or DCE	26	ERC	1.5/3T
Panebianco et al. 2010	DCE, MRSI, MRSI and DCE	150	ERC	1.5T
Perrotti et al. 2002	T2	74	ERC	1.5/3T
Prando et al. 2005	T2, MRSI, T2 and MRSI	42	ERC	1.5T
Sciarra et al. 2010	DCE, MRSI, MRSI and DCE	90	ERC	1.5T
Tamada et al. 2011	T2, DCE, T2 or DWI or DCE	50	PAC	1.5T
Tanimoto et al. 2007	T2 or DWI or DCE	83	PAC	1.5T
Testa et al. 2010	T2, MRSI, T2 and MRSI, T2 or MRSI	54	ERC	1.5T
Vilanova et al. 2011	T2 or DWI or DCE	140	ERC	1.5T
Wetter et al. 2005	T2, MRSI, T2 and MRSI, T2 or MRSI	6	ERC	1.5T
Yao et al. 2009	T2	41	ERC	3T
Younes et al. 2001	T2	27	ERC	1.5T
Yuen et al. 2004	T2, MRSI, T2 and MRSI, T2 or MRSI	24	ERC	1.5T
Petrillo et al. 2014	T2, MRSI, T2 and MRSI	136	ERC	1.5T
Petrillo et al. 2013	MRSI and DCE	106	ERC	1.5T
Heijmink et al. 2010	T2	46	PAC	3T
Sciarra et al. 2007	T2, DCE, MRSI and DCE	50	ERC	1.5T

*ERC* endorectal coil, *PAC* phase array coil

22 studies [12, 17, 41, 44, 45, 52, 61–76] involving 2294 patients reported the diagnostic accuracy of T2-MRI. 8 studies [17, 43, 52, 65, 68, 75–77] involving 1445 patients reported the diagnostic accuracy of DCE-MRI. 10 studies [12, 43–45, 61, 64, 66, 70, 73, 77] involving 618 patients reported the diagnostic accuracy of MRSI. 5 studies [52, 53, 65, 67, 68] involving 313 patients reported the diagnostic accuracy of T2-MRI combined to DCE-MRI. 5 studies [76, 78–81] involving 2121 patients reported the diagnostic accuracy of T2-MRI combined to DWI and DCE-MRI. 9 studies [44, 45, 61, 64, 66, 67, 70, 73, 82] involving 407 patients reported the diagnostic accuracy of T2-MRI combined to MRSI. 4 studies [2, 43, 75, 77] involving 394 patients reported the diagnostic accuracy of MRSI combined to DCE-MRI. Only 2 studies (Petrillo et al. [12] and Haider et al. [41]) reported the diagnostic accuracy of T2-MRI combined with DWI with some discrepancies in the results: sensitivity of 64% and 81%, specificity of 46% and 84%, positive predictive value of 21% and 88% and negative predictive value of 89% and 83%, respectively. One study [76] reporting the findings of DW-MRI alone was found (sensitivity and specificity of 87% and 92%, respectively).

**Fig. 5:**
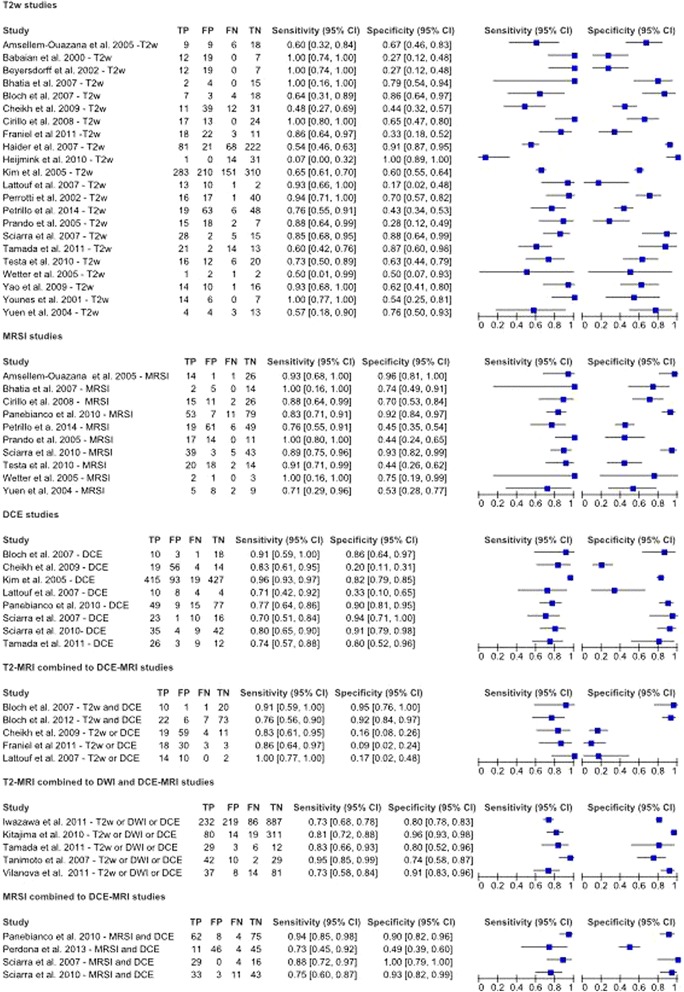
Forest Plot including sensitivity, specificity estimates and their confidence intervals (95%) for each MRI modality and their combinations in a multiparametric MRI approach

**Fig. 6:**
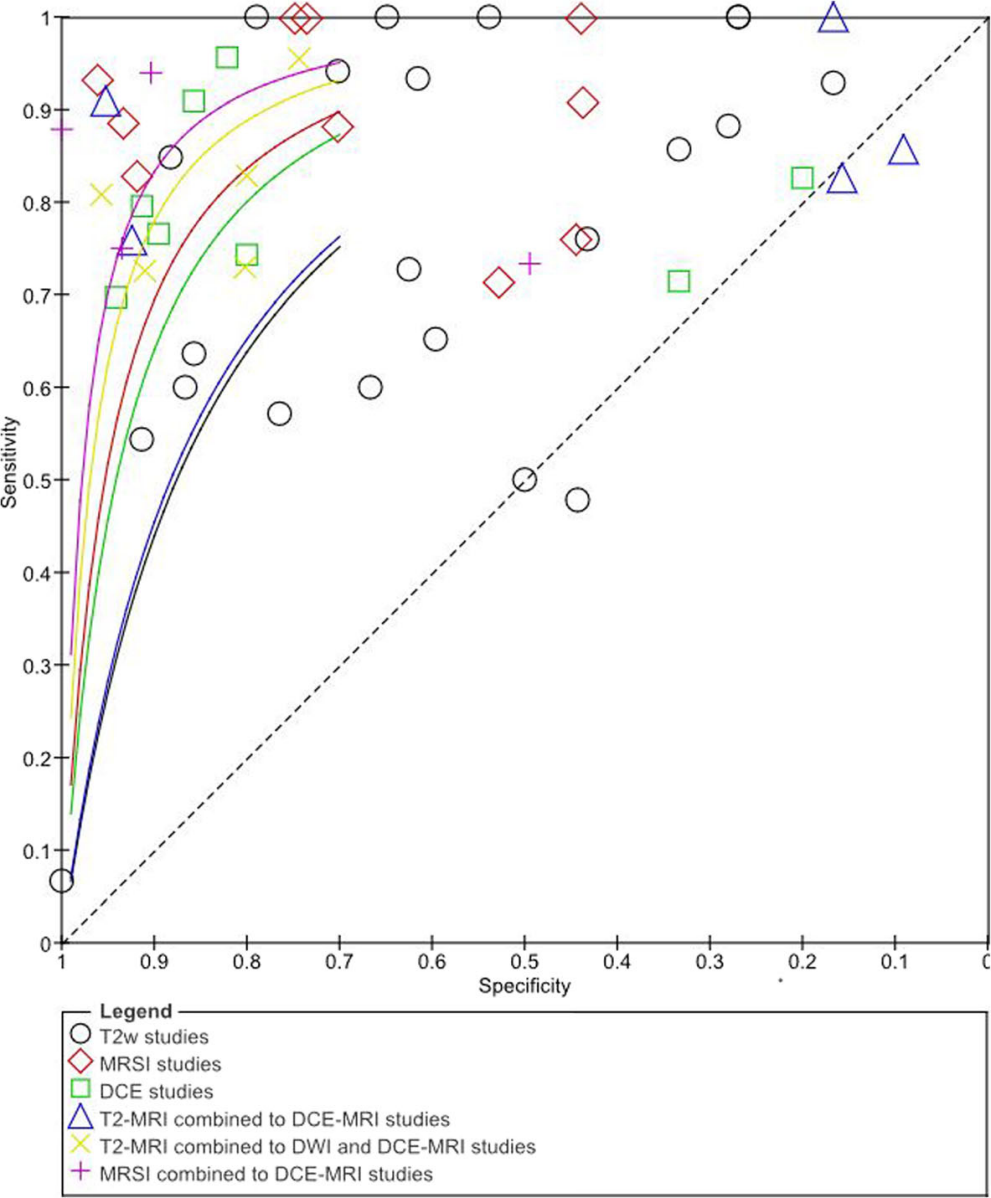
Estimated Summary ROC curves and original data points for each MRI modality and their combinations in a multiparametric MRI approach

Figure 5 reports the values of TP (True Positive), FP (False Positive), FN (False Negative), TN (True Negative), sensitivity and specificity estimates and their confidence intervals (95%) for each study; moreover, the figure reports in the name of the study whether the modalities combination is considered as “OR” or “AND”. Figure 6 shows ROC for each diagnostic modality and their combinations. For the T2-MRI, we had a sensitivity of 75% and specificity of 60%; for the DCE-MRI, the sensitivity was 80% and the specificity was 72%; for the MRSI, the sensitivity was 89% and specificity was 69%; for the combination T2-MRI and DCE-MRI, the sensitivity was 87% and specificity was 46%; for the combination T2-MRI and MRSI, the sensitivity was 79% and specificity was 57%; for the combination T2-MRI, DWI and DCE-MRI, the sensitivity was 81% and specificity was 84%; and for the combination MRSI and DCE-MRI, the sensitivity was 83% and specificity was 83% (Table 3).

**Table 3:** Performance pooled analysis for MRI, DWI, DCE-MRI, PET/CT and multimodal imaging

Performance Pooled Analysis	Sensitivity	Specificity	PPV	NPV	ACC
T2-MRI	75,12	59,82	55,38	81,51	65,21
DCE-MRI	80,08	72,01	74,90	75,37	73,58
MRSI	89,14	68,58	59,71	92,49	74,81
T2-MRI combined with DCE-MRI	87,02	45,82	57,93	81,96	62,88
T2-MRI combined with MRSI	78,76	57,13	52,64	84,46	63,39
T2-MRI combined with DWI and DCE - MRI	80,92	84,25	78,03	86,18	84,53
MRSI combined with DCE - MRI	82,54	83,32	74,88	86,60	80,26

**Fig. 7:**
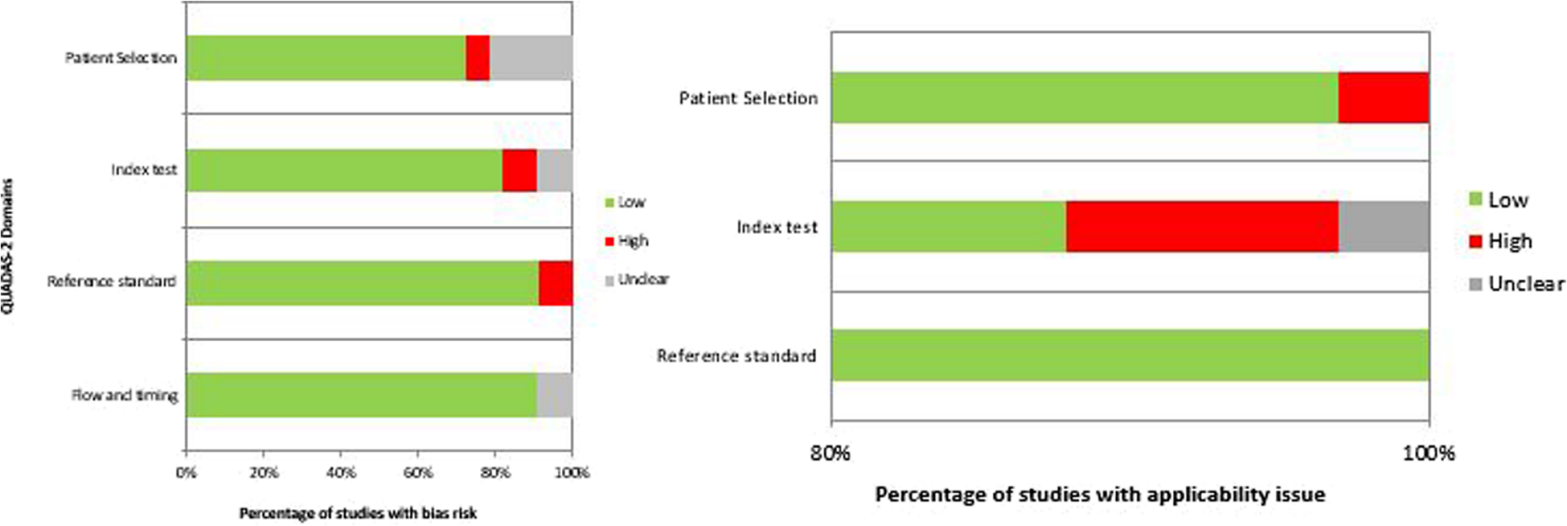
Studies quality analysis by means QUADAS-2 domains

Overall, the quality was moderate (Fig. 7). In particular, the bias risk was unclear for some studies due to the lack of reporting on patient enrolment, on blinding to the index test during evaluation of the reference test. In the patient selection sphere, few studies had a high risk of bias because of inappropriate exclusion criteria. For the index test domain, high risk of bias was associated at the studies that did not provide a cut-off level and /or the readers were not blinded to the reference test. For the reference test domain, high risk of bias was associated at the studies where the reference standard was interpreted without blinding to the index test. No studies were excluded from the analysis based on the quality assessment.

**Fig. 8:**
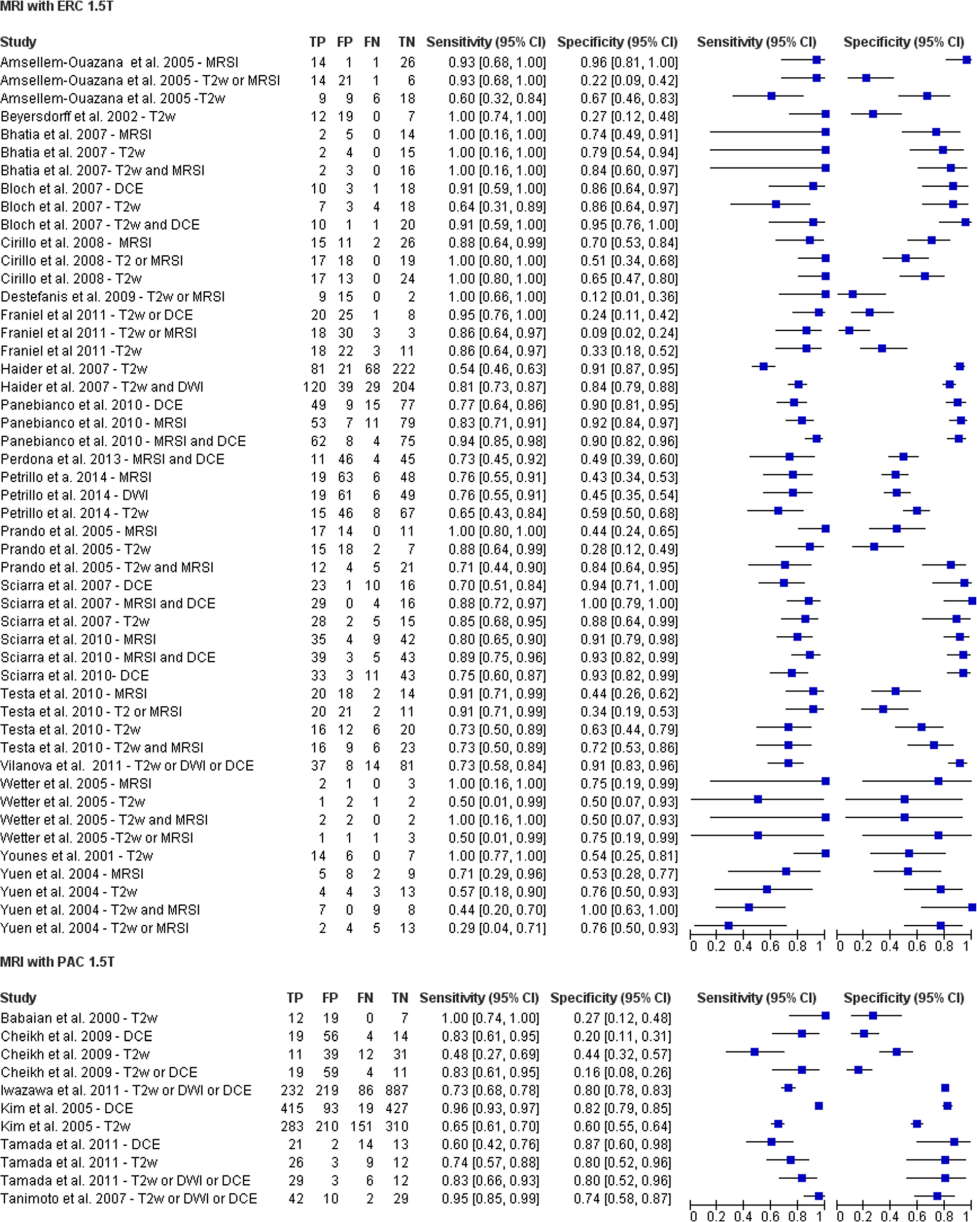
Forest Plot including sensitivity, specificity estimates and their confidence intervals (95%) for MRI acquired with ERC versus MRI acquired with PAC

**Fig. 9:**
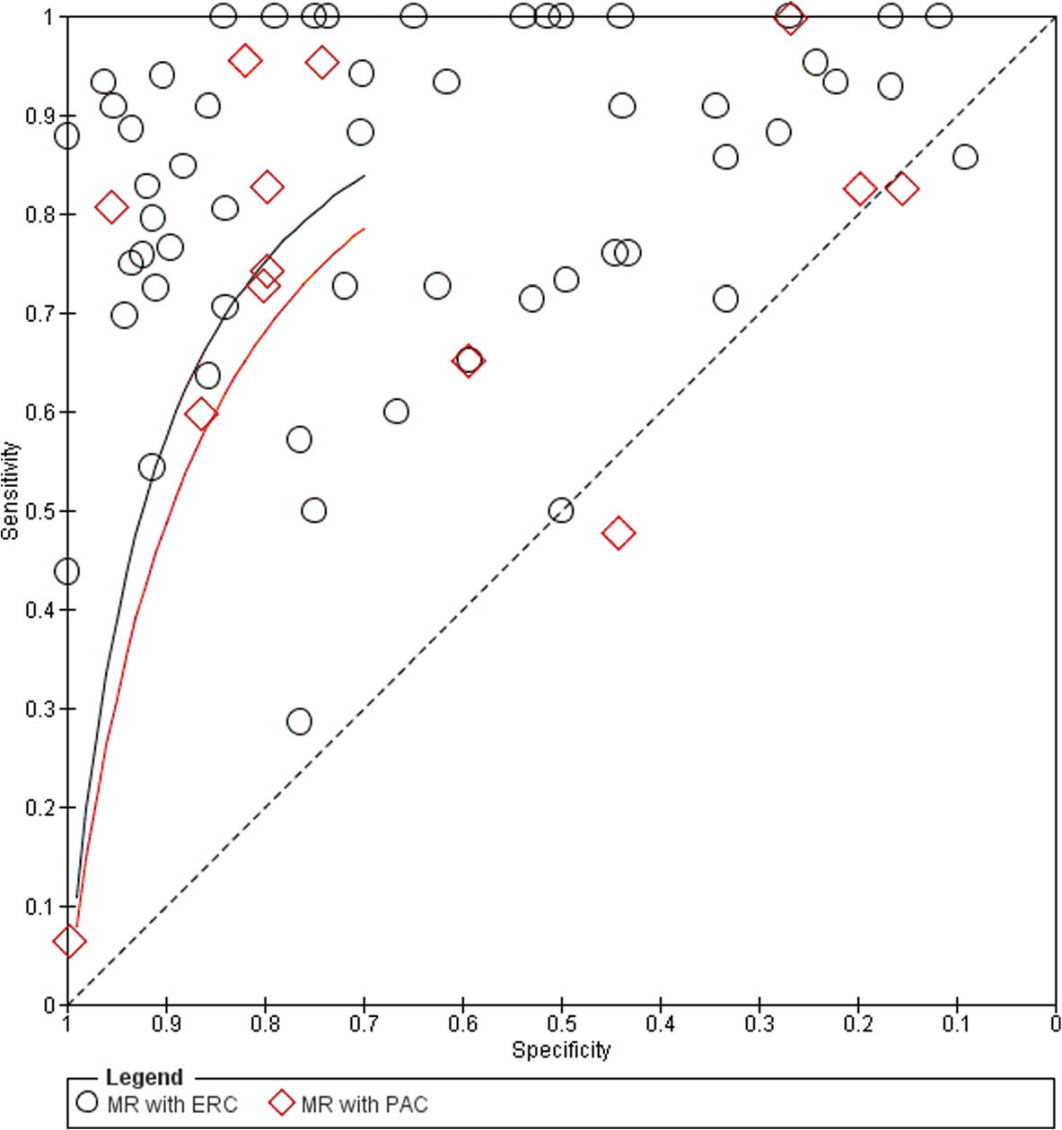
Estimated summary ROC curves and original data points for MRI acquired with ERC versus MRI acquired with PAC

Figure 8 shows Forest plot and Fig. 9 shows ROC curves for the use of ERC for 3467 patients versus the use of PAC for 3882 patients in the MRI acquisition using 1.5T scanner. For MRI studies that utilized ERC we obtained a pooled sensitivity, specificity, positive, negative predictive and accuracy value of 81%, 66%, 60% and 86% and 71%, respectively. For MRI studies that utilized PAC we obtained a pooled sensitivity, specificity, positive, negative predictive value and accuracy of 78%, 64%, 61% and 82% and 68%, respectively. The use of ERC did not increase significantly the diagnostic accuracy in prostate cancer detection (*p* > 0.05 at McNemar test).

## Discussions and Conclusions

In this review, we collected the current evidence of the role of morphological T2-MRI and functional MRI modalities (DCE-MRI, DWI and MRSI) in the prostate cancer detection.

Among the 22 studies reporting the accuracy of T2-MRI, 5 studies showed a sensitivity of 60% or lower. In addition, 4 studies reported specificity of 35% or lower for T2-MRI. Moreover, as reported by Roethke et al. [22] prostate cancer detection depends by tumour size: T2-MRI cannot exclude prostate cancer with lesions smaller than 10 mm (0.4 cm^3^), in this case, prostate cancer detection is lower than 13%. Instead, the detection rate for lesions more than 20 mm (1.6 cm^3^) was higher (45-89%).

MRSI studies reported the Cho-Cr/Ci ratio used as the cut-off for a positive test result, which ranged from > 0.6 to > 0.86. All of the studies demonstrated a sensitivity of ≥ 83% apart from Petrillo et al. [12] and Yuen et al. [73] that reported a sensitivity of 76 and 71%, respectively. Petrillo et al. [12] and Yuen et al. [73] suggested that influential factors to the low sensitivity reported might have been difficulties in ensuring the correspondence of TRUS biopsy spatial accuracies to suspicious areas on MRI. Moreover, the studies by Petrillo et al. [12], Prando et al. [44] and Testa et al. [45] reported low specificity (44-46%). Testa et al. [45] suggested that the low specificity in their study was probably determined by the lower Cho+Cr/Ci ratio used (actual value not reported) compared with cut-offs used by other studies.

Concerning to DCE-MRI, Engelbrecht et al. [28] reported the usefulness of relative peak enhancement and washout rate for prostate cancer detection and localization in the peripheral zone and gland central region (areas under the receiver operating characteristic curve were 0.93 and 0.82, respectively). Kim et al. [19] demonstrated, instead, that the wash-in rate was more accurate for the detection of prostate cancer in the peripheral zone (wash-in rate sensitivity and specificity of peripheral zone cancer detection were 96% and 97%). However, they also observed significant overlap between the wash-in rate for cancer and normal tissue in the transitional zone. Moreover, some limitations of this modality were reported such as the inadequate depiction of transitional zone cancer in patients with hypervascular benign prostatic hyperplasia. Hoeks et al. [20] reported that DCE-MRI did not show additional benefits compared to T2-MRI for detection of cancer in the transition zone. Instead, compared with the other DCE-MRI studies, the study by Sciarra et al. [75] reported high specificity (91%).

In our analysis, the use of functional techniques in addition to T2-MRI appeared to have a large influence on sensitivity. In fact, ROC curves demonstrated that Multimodal Imaging combining MRSI and DCE-MRI or combining T2-MRI, DWI and DCE-MRI had the best accuracy in term of sensitivity and specificity (Fig. 7, Table 3). A multiparametric approach combining MRSI and DCE-MRI reached an increase of sensitivity of 8% and an increase of specificity of 23% while combining T2-MRI, DWI and DCE-MRI we obtained an increase in sensitivity and specificity of 6% and 24% respectively, compared to the morphological T2-MRI alone. Panebianco et al. [43] demonstrated that the combination of MRSI and DCE-MRI yielded 93.7% of sensitivity, 90.7% of specificity, 88.2% of positive predictive value, 95.1% of negative predictive value and 90.9% of accuracy in detecting prostate carcinoma. Similar results were provided by Petrillo et al., Perdonà et al. [12, 13] and Sciarra et al. [75].

Fusco et al. [83] in a recent study showed that combining morphological MRI, DWI, DCE-MRI and MRSI, an increase in sensitivity and specificity correlated to biopsy Gleason grade was obtained.

Our results are comparable with other review and meta-analysis [84–87]. Results of the Rooij et al. [87] meta-analysis suggested that T2-MRI with DWI and DCE-MRI is the best combination to provide better characterization of tumour in the prostate with a high overall sensitivity and specificity of 74% and 88%, respectively, and negative predictive value ranging from 65% to 94%. In another study [88], multiparametric MRI showed good performance at detecting and ruling out clinically significant cancer, following at least one previous biopsy, with a negative predictive value of 95% using transperineal template systemic biopsy as the gold standard. The authors concluded that multiparametric MRI can therefore be used as a triage test following a negative biopsy and thereby identify patients who can avoid further biopsies.

Because prostate MRI interpretation can be subjective and inconsistent, suspicion scores for prostate cancer on MRI (Prostate Imaging and Reporting Archiving Data System [PI-RADS]) have been recently developed on a 1- to 5-point scale for improved standardization of MRI interpretation and reporting [89, 90] using a multiparametric approach with DCE-MRI, DWI and MRSI. A recent meta-analysis of 14 studies evaluating use of the PI-RADS scoring system for prostate cancer detection on multiparametric MRI showed good diagnostic accuracy [89]. These studies are not inserted in this systematic review because the PI-RADS scoring system is work in progress and PI-RADS version 2 has recently been published [90].

We also evaluated the differences in the diagnostic accuracy for prostate cancer detection between the use of ERC and PAC in MRI setting. Generally, use of a higher field strength (3.0T instead of 1.5T) or the use of endorectal coil improved the detection sensitivity for extracapsular extension (ECE) and seminal vesicle invasion (SVI) detection [91, 92]. ERC should be used for a field strength of 1.5T in the absence of multiparametric MRI. ERC is useful for its capability to increase image resolution and to improve staging accuracy [91, 92]. Costa et al. [92] showed that the use of combined ERC and PAC for T2-MRI and DWI with 3T Magnetic Resonance scanner provides superior sensitivity for the detection of prostate cancer (78%) compared to an examination performed without the ERC (43%). Different results were reported from Baur et al. [93]: T2-MRI and DWI had a range of area under the curve with a PAC and with ERC-PAC of 0.95-0.99 and 0.93-0.97, respectively. They concluded that T2-MRI and DWI performed at 3T for prostate cancer lesion identification and evaluation did not differ significantly with both coil setups and that patients preferred MRI without an ERC. Moreover, the ERC leads to deformity in the prostate contour, and the anatomical distortion resulting from it can potentially hinder the diagnosis and pathology correlation [94]. Another limitation is that patients with rectal stenosis or immediately after surgery or radiotherapy may not be good candidates for the use of the ERC during MRI examination. When higher field strengths or multichannel (8 channel or more) PAC and additional functional techniques were used, studies that used an ERC showed lower sensitivity and heterogeneous specificity than studies without an ERC [84]. Lee et al. [16] reported that the use of ERC in MRI acquisition did not significantly improve the staging of prostate cancer (AUC = 0.67 versus 0.66 respectively with and without ERC) and presented several complications in 11.4% of patients. Margolis et al. [95] reported that an ERC is not absolutely necessary and that the utility will depend on the performance of the scanner in question. Therefore, the use of multi-channel PAC during MRI acquisition could be an alternative considering comorbidity and could replace the use of an ERC. Also the European Society of Urogenital Radiology prostate MR guidelines reported in acquisition protocols minimum requirements that MRI can adequately be performed at 1.5 T using a good 8- to 16 channels PAC [96]. In our systematic review, we demonstrated that ERC yielded no additional benefit for the detection of prostate cancer: there was no statistically significant increase (*p* > 0.05 at McNemar test) in sensitivity (81% versus 78%) and specificity (66% versus 64%). On the other side, the use of functional MRI in a multiparametric approach (MRSI and DCE-MRI or T2-MRI, DWI and DCE-MRI) improves the accuracy in prostate cancer detection allowing both the early cure and the guidance of biopsy. MRSI combined to DCE-MRI reached an increase of sensitivity of 8% and an increase of specificity of 23% while combining T2-MRI, DWI and DCE-MRI we obtained an increase in sensitivity and specificity of 6% and 24%, respectively.

About the limitations of this study: most papers reported on a limited number of patients. Because of the heterogeneity within the included studies with respect to patient selection, imaging protocols and analyses, this pooled analysis should be regarded as an indicator of the general performance of morphological T2-MRI and functional MRI in a multiparametric approach for prostate cancer detection and localization.
